# Prevalence of antibiotic resistance in lactic acid bacteria isolated from the faeces of broiler chicken in Malaysia

**DOI:** 10.1186/1757-4749-6-1

**Published:** 2014-01-22

**Authors:** Nurhazirah Shazali, Hooi Ling Foo, Teck Chwen Loh, Di Wei Choe, Raha Abdul Rahim

**Affiliations:** 1 Department of Animal Science, Faculty of Agriculture, Universiti Putra Malaysia, 43400, UPM Serdang Selangor, Malaysia; 2 Department of Bioprocess Technology, Faculty of Biotechnology and Biomolecular Sciences, Universiti Putra Malaysia, 43400, UPM Serdang Selangor, Malaysia; 3 Department of Cell and Molecular Biology, Faculty of Biotechnology and Biomolecular Sciences, Universiti Putra Malaysia, 43400, UPM Serdang Selangor, Malaysia; 4 Institute of Bioscience, Universiti Putra Malaysia, 43400, UPM Serdang Selangor, Malaysia; 5 Institute of Tropical Agriculture, Universiti Putra Malaysia, 43400, UPM Serdang Selangor, Malaysia

**Keywords:** Lactic acid bacteria, Antibiotic resistance, Broiler chicken

## Abstract

**Background:**

Probiotics are commonly used as feed additive to substitute antibiotic as growth promoter in animal farming. Probiotic consists of lactic acid bacteria (LAB), which enhance the growth and health of the animal. Probiotic also have higher possibility to become pathogenic bacteria that may carry antibiotic resistant gene that can be transmitted to other LAB species. The aim of this study was to identify the LAB species in the faeces of broiler chicken and to determine the prevalence of antibiotic resistant in LAB of broiler chicken.

**Methods:**

Sixty faeces samples were collected from wet markets located in Klang Valley of Malaysia for the isolation of LAB using de-Mann Rogosa Sharpe medium. Thirteen species of LAB were obtained in this study and the identification of LAB was performed by using API test kit on the basis of carbohydrate fermentation profile. Antibiotic susceptibility assay was then carried out to determine the prevalence of LAB antibiotic resistance.

**Results:**

*Lactococcus lactis* subsp *lactis* was found in nine out of sixty faecal samples. *Lactobacillus paracasei* was the second common LAB species isolated from chicken faecal. No significant difference (P > 0.05) was found between the occurrence of *Lactobacillus brevis, Lactobacillus curvatus, Lactobacillus plantarum, Leuconostoc lactis mesenteroides* subsp *mesenteroides*/*dectranium* and *Pediococcus pentosaceus* isolated from 5 different locations. Most of the isolated LAB was resistant to antibiotic and high variability of the antibiotic resistance was observed among the LAB against 15 types of antibiotics. Penicillin, amoxicillin, chloramphenicol, and ampicillin had significant higher (*P*< 0.05) inhibitory zone than nalidixic acid, gentamycin, sulphamethoxazole, kanamycin, and streptomycin.

**Conclusions:**

Many species of LAB were isolated from the faecal samples of broiler chicken that resistance to the common antibiotics used in the farm. The development of resistant against antibiotics in LAB can be attributed to the long term exposure of antibiotic as growth promoter and therapeutic agents. Thus, it is essential to advise farmer the safety measure of antibiotic application in animal farming. Additionally, the supplementation of probiotic in animal feeding also needs more attention and close monitoring.

## Background

Antibiotic are normally used to treat microbial diseases since 50 years ago. However, excessive use of antibiotic may lead to the development of antibiotic resistance in pathogenic bacteria. The report of antibiotic resistance is significantly increased due to the overuse and misuse of antibiotics, which has created an enormous selective pressure on the recessive bacteria [1]. Antibiotic resistant bacteria have the ability to resist toward the actions of naturally occurring or synthetically produced compounds inimical to their survival [2].

Scott [3] reported the identical resistance gene present in bacterial species isolated from different hosts. Antibiotic resistant may acquires through the *in-vivo* gene transfer between normal flora of gastrointestinal and antibiotic resistant pathogenic bacteria [4]. In fact, the antibiotic resistant pathogenic bacteria pose a great potential threat to human health, especially when the immunity system is not functioning well. Most of the developed countries have prohibited the use of antibiotic as animal growth promoter. Thus, various alternatives have been explored to replace antibiotic as growth promoter. One of the most common and popular alternatives is the application of probiotic as growth promoter in livestock animals. In addition, the probiotic effects of postbiotic metabolites produced by probiotic strains have been shown in various animal species, such as rats [5,6], broilers [7,8], laying hens [9] and post weaning piglets [10], suggesting they have great potential to be used as growth promoter for livestock animals.

Probiotic comprises of beneficial bacteria such as lactic acid bacteria (LAB). LAB is a group of gram-positive anaerobic bacteria which produce predominantly lactic acid from carbohydrate fermentation. Many farmers use probiotic extensively and those bacteria have potential to serve as a host of antibiotic resistance genes with a risk of transferring those genes into many LAB and other pathogens [11].

The antibiotic resistant LAB has been detected by using DNA sequences which is responsible for antibiotic resistance traits. Egervärn *et al.*[12] reported the emergence of antibiotic resistant in *Lactobacillus reuteri* and *Lactobacillus plantarum*. Moreover, *Lactobacilli*, *Pediococci* and *Leuconostoc* spp*.* have been reported to be highly resistant to vancomycin [11,13] and some *Lactobacilli* have high resistance to bacitracin, cefoxitin, ciprofloxacin, fusidic acid, streptomycin, sulphadiazine, teicoplanin and vancomycin [14]. Most of the reported LAB that resistant to antibiotics was isolated from food sources. These include the most commonly used probiotic species such as *Lactobacillus casei*, *Lactobacillus acidophilus*, *L. reuteri*, or *Lactobacillus rhamnosus*, among others, or the yogurt starter bacteria *Lactobacillus delbrueckii*[15-17]. However, the information of antibiotic resistant LAB isolated from the gut of broiler chickens in Malaysia is very limited. Thus, the objective of this study was to investigate the antibiotic resistance profile of LAB isolated from the gut of broiler chickens in Klang Valley, Malaysia.

## Materials and methods

### Sample origin and collection

A total of 60 faeces samples were collected from chicken purchased from wet markets located nearby Universiti Putra Malaysia: Serdang Jaya, Seri Kembangan, Dengkil, Putrajaya, Kajang, Pantai Dalam, and Jenderam Hilir. The age of the chicken was 42 days. The faeces were collected directly from caeca junction to the end of the cloaca of large intestine. The faeces samples (1 g each) were kept at -20°C until further analysis. The experimental design has obtained approval from the Ethics Committee of Universiti Putra Malaysia.

### Enumeration and isolation of lactic acid bacteria

The faeces sample was mixed homogeneously at a ratio of 1 g sample with 9 ml of peptone water in the universal bottle and incubated for one hour at room temperature. The samples were then subjected to 10-fold serial dilution using 0.1% (v/v) peptone water [18] and 0.1 ml of each appropriate diluted sample was then plated onto de Man Rogosa Sharpe (MRS) agar and incubated under anaerobic condition at 30°C for 48 hours [19]. The colony forming unit (CFU) per gram of sample was expressed as logarithm at the base of 10 (Log_10_CFU/g). The enumeration of LAB was conducted in triplicates. After 48 hours of incubation, a colony was picked and streaked onto MRS agar and incubated for additional 48 h at 30°C. This process was repeated twice and a pure colony was then transferred to MRS broth and incubated at 30°C for 24 h. The pure LAB culture was then kept in MRS broth supplemented with 20% (v/v) glycerol and incubated at -20°C until further analysis.

### Identification of lactic acid bacteria

LAB culture was identified phenotypically on the basis of carbohydrate fermentation profile by using API 50CH kit (BioMerieux, France) according to the instruction of manufacturer. The carbohydrate fermentation profile was then analysed by using APILAB Plus software version 3.3.3 (BioMerieux, France) to identify the species of each isolated LAB culture.

### Antibiotic susceptibility assay

The antibiotics used for susceptibility assay were ampicillin (10 μg), clindamycin (2 μg), erythromycin (15 μg), gentamicin (10 μg), streptomycin (25 μg), tetracycline (30 μg), chloramphenicol (30 μg), kanamycin (30 μg), sulphamethoxazole/trimethoprim (25 μg), vancomycin (30 μg), ciprofloxacin (5 μg), amoxicillin (10 μg), bacitracin (10 μg), nalidixic acid (30 μg), and penicillin (10 μg) (Oxoid Ltd, England). The antibiotics were selected due to their common use in local animal farming. A total of 1 ml LAB culture grown in MRS broth was collected by centrifugation at 1000 × g for 5 min. The cell pellet was collected and washed twice using 1 ml of 0.85% (w/v) NaCl, followed by suspending the cell pellet with 0.5 ml of 0.85% (w/v) NaCl. The cell suspension was adjusted to 0.5 Mc Farland by using 2 ml of NaCl 0.85% (w/v) prior to spread plate on MRS agar. The antibiotic disc was then placed on MRS agar plate. The diameter of inhibitory zones was measured after 48 h of incubation at 30°C under anaerobic condition. The assay was conducted in triplicates [20].

### Statistical analysis

The data was analysed using one way analysis of variance (ANOVA) with probability level of 0.05 (P < 0.05) using SAS statistical software [21].

## Results and discussion

### Identification of lactic acid bacteria

All LAB species isolated from faeces of broiler chicken were identified by API Kit. Table 1 shows the identity of LAB species isolated from various wet markets located in Klang Valley of Malaysia. The result demonstrated that different LAB species were isolated from different wet market and the most common LAB species isolated from chicken faecal was *Lactococcus lactis* subsp *lactis* as it was isolated from 9 out of 60 samples. *Lactobacillus paracasei* was the second common LAB species isolated from chicken faecal. However, there was no significant different (P > 0.05) between the occurrence of *Lactobacillus brevis, Lactobacillus curvatus, Lactobacillus plantarum, Leuconostoc lactis mesenteroides* subsp *mesenteroides*/*dectranium* and *Pediococcus pentosaceus* as they were isolated from 5 different locations. *Pediococcus damnosus* was isolated from 4 locations, whereas *Lactobacillus fermentum* and *L. rhamnosus* (*Lactobacillus casei* subsp *rhamnosus*) were isolated from 2 locations.

**Table 1:** Lactic acid bacteria species isolated from faecal samples collected from various locations

**Locations**	***L. acidophilus***	***L. brevis***	***L. curvatus***	***L. delbrueckii *** **subsp *delbrueckii***	***L. fermentum***	***L. paracasei *** **subsp *paracasei***	***L. plantarum***	***L. rhamnosus (L. casei *** **subsp *rhamnosus)***	***L. salivarius***	***Lc. lactis *** **subsp *lactis***	***Leu. lactis mesenteroides *** **subsp *mesenteroides/dextranicum***	***P. damnosus***	***P. pentosaceus***
Dengkil 1	0	0	1	0	0	3	0	0	0	1	0	0	1
Dengkil 2	0	0	2	0	0	0	0	1	0	1	0	2	0
Jenderam Hilir	0	0	1	3	0	0	0	0	0	1	0	0	1
Kajang	0	2	0	0	0	0	0	0	0	2	2	0	0
Serdang Jaya 1	0	0	1	0	0	0	1	0	0	2	0	2	0
Serdang Jaya 2	0	2	0	2	1	1	0	0	0	0	0	0	0
Seri Kembangan	1	0	0	1	0	3	0	1	0	0	0	0	0
Pantai Dalam	0	0	0	0	1	0	1	0	2	0	2	0	0
Putrajaya	0	1	0	0	0	0	2	0	1	2	0	0	0
UPM	0	0	0	0	0	0	1	0	1	0	1	0	3
**Total**	**1**	**5**	**5**	**6**	**2**	**7**	**5**	**2**	**4**	**9**	**5**	**4**	**5**

Note: A total of 6 samples were collected from each location.

It has been reported that *L. acidophilus* and *Lactobacillus salivarius* have higher chance to be isolated among the *Lactobacillus* strains colonizing in the crop intestine [22]. However, *L. acidophilus* and *L. salivarius* were only found in faeces samples 1 and 4, respectively; indicating *L. acidophilus and L. salivarius* were not a common LAB found in the faeces of chicken sold in Klang valley of Malaysia.

### Antibiotic resistance of lactic acid bacteria

Table 2 shows the inhibitory zone of antibiotic susceptibility for nine LAB regardless of species obtained from faecal samples. Penicillin, amoxicillin, chloramphenicol, and ampicillin had significant higher (P < 0.05) inhibitory zone than nalidixic acid, gentamycin, sulphamethoxazole, kanamycin, and streptomycin. These results were similar with the previous report, where lactobacilli were most sensitive to penicillin and ampicillin except for amoxicillin and chloramphenicol [23]. However, there was no significantly different (P > 0.05) between vancomycin and bacitracin. Similar inhibitory zone was also found in bacitracin, nalidixic acid, gentamycin, sulphamethoxazole, kanamycin, and streptomycin. LAB was sensitive to clindamycin, chloramphenicol, amoxicillin, penicillin, erythromycin, and ampicillin in current study. However, it was resistant against nalidixic acid, bacitracin, gentamycin, ciprofloxacin, sulphamethoxazole, kanamycin, tetracycline, streptomycin and vancomycin. These results indicate that LAB is not sensitive to beta-lactams group of antibiotics due to the absence of peptidoglycan in LAB cell. However, Lavanya *et al.*[24] reported recently that most of the LAB isolated from fermented milk were resistant to penicillin G and only 10% were susceptible to ampicillin. Beta-lactams antibiotics were the most effective drugs for the treatment of *Staphylococci* infections. However, Tyler *et al.*, [25] reported that beta-lactams antibiotics have no longer effective to treat *Staphylococcus aureus* infections such as methicillin-resistant *S. aureus* and vancomycin-resistant *S. aureus.*

**Table 2:** Diameter inhibitory zone (Mean ± SE), cm of antibiotic susceptibility test for LAB regardless of species from chicken faecal sample

**Antibiotics**	**Diameter of inhibitory zone (cm)**
**Nalidixic acid**	0.02^e^ ± 0.02
**Clindamycin**	1.22^b^ ± 0.16
**Bacitracin**	0.33^de^ ± 0.07
**Chloramphenicol**	1.89^a^ ± 0.17
**Amoxycillin**	1.93^a^ ± 0.16
**Gentamycin**	0.07^e^ ± 0.04
**Ciprofloxacin**	0.21d^e^ ± 0.06
**Penicillin**	1.99^a^ ± 0.17
**Sulphamethoxazole**	0.11^e^ ± 0.05
**Kanamycin**	0.05^e^ ± 0.04
**Tetracycline**	0.75^c^ ± 0.13
**Erytromycin**	1.17^b^ ± 0.15
**Ampicillin**	1.84^a^ ± 0.16
**Streptomycin**	0.05^e^ ± 0.04
**Vancomycin**	0.51^cd^ ± 0.12

Note: ^a-e^Means with different superscripts are significantly different ( P< 0.05).

The development of antibiotic resistance in LAB has revealed the fact that the common use of antibiotic as growth promoter in the farm has allowed LAB to develop resistance against antibiotics [26]. The antibiotic resistance in LAB may also acquire through horizontal transferring of resistance gene to the normal LAB by conjugative transposons [1]. As reported by Gilchrist *et al. *[26], antibiotic resistance in LAB was partly due to poor control of antibiotic as therapeutic and growth promoter in animal feeding. This may contribute to the build-up reservoir of antibiotic resistant bacteria in the gut. Hence, the current result suggests that antibiotic resistance screening is essential before any LAB strains are selected as feed additive for animal feeding.

### Profile of lactic acid bacteria antibiotic resistance

Table 3 shows the diameter of inhibitory zone of antibiotic susceptibility among the LAB species. The inhibitory zone for *Leu. lactis mesenteroides* subsp *mesenteroides/dextranicum* was significantly higher (P < 0.05) than other LAB species for the tested antibiotics. Bassam [27] reported that *Leu. lactis mesenteroides* was resistant to 100% vancomycin, 75% teicoplanin, 87.5% clindamycin, 75% chloramphenicol, erythromycin, gentamicin and streptomycin, 62.5% ampicillin, penicillin G and tetracycline, 50% kanamycin and trimethoprim. *L. acidophilus* was resistant (P < 0.05) against nalidixic acid, gentamycin, ciprofloxacin, sulphamethoxazole, kanamycin, streptomycin and vancomycin. However, Sieo *et al.*[28] claimed that *L. acidophilus* isolated from chicken gastrointestinal tract was resistant to 100% chloramphenicol (≥200 μg/ ml), 58% to erythromycin (≥200 μg/ml) and 17% to tetracycline (≥200 μg/ml). *L. brevis and L. curvatus* had similar trend of resistance against the antibiotics. However, *L. brevis* was more resistant to ciprofloxacin (a fluoroquinolone), tetracycline, and vancomycin as found in the study of Fukao *et al.*[29]. *L. delbrueckii* subsp *delbrueckii, L. fermentum, L. rhaminosus, L. salivarious, Leu. mesenteroides, P. damnosus, P. pentosaceus* were sensitive to all the tested antibiotics.

**Table 3:** Diameter, cm (Mean ± SE) of inhibitory zone of lactic acid bacteria species

**Lactic acid bacteria**	**Sulphamethoxazole**	**Kanamycin**	**Tetracycline**	**Erytromycin**	**Ampicillin**	**Streptomycin**	**Vancomycin**
***L. acidophilus***	0.00^a^±0.00	0.00^a^±0.00	1.28^a^±0.16	1.22^a^±0.16	1.22^a^±0.16	0.00^a^±0.00	0.00^a^±0.00
***L. brevis***	0.00^c^±0.00	0.00^c^±0.00	0.60^abc^±0.08	1.12^abc^±0.15	2.02^ab^±0.26	0.00^c^±0.00	0.50^bc^±0.06
***L. curvatus***	0.00^a^±0.00	0.00^a^±0.00	0.00^a^±0.00	0.60^a^±0.08	1.40^a^±0.18	0.00^a^±0.00	0.52^a^±0.07
***L. delbrueckii *** **subsp *delbrueckii***	19.75^a^±2.54	22.00^a^±2.84	24.25^a^±3.13	26.50^a^±3.42	29.30^a^±3.78	31.00^a^±4.00	33.78^a^±4.36
***L. fermentum***	2.25^a^±0.29	2.50^a^±0.03	3.08^a^±39	3.62^a^±0.47	4.68^a^±0.60	3.50^a^±0.45	4.40^a^±0.57
***L. paracasei *** **subsp *paracasei***	0.00^g^±0.00	0.28^fg^±0.04	1.30^cdef^±0.17	1.88^abcd^±0.24	2.60^ab^±0.34	0.22^fg^±0.03	1.00^defg^±0.13
***L. plantarum***	0.00^f^±0.00	0.00^f^±0.00	1.80^abcd^±0.23	1.58^abcd^±0.20	2.50^ab^±0.32	0.00^f^±0.00	1.15^cdef^±0.15
***L. rhamnosus (L. casei *** **subsp *rhamnosus)***	20.00^a^±2.58	22.25^a^±2.87	25.15^a^±3.25	27.38^a^±3.53	30.02^a^±3.88	31.25^a^±4.03	33.50^a^±4.33
***L. salivarius***	2.25^a^±0.29	3.00^a^±0.39	2.75^a^±0.36	3.45^a^±0.45	4.22^a^±0.55	3.50^a^±0.45	4.25^a^±0.55
***Lc. Lactis *** **subsp *lactis***	0.25^b^±0.03	0.00^b^±0.00	0.78^b^±0.10	1.92^a^±0.25	2.35^a^±0.30	0.00^b^±0.00	0.48^b^±0.06
***Leu. lactis mesenteroides *** **subsp *mesenteroides/ dextranicum***	39.5^a^±5.10	44.00^a^±5.68	48.50^a^±6.26	53.65^a^±6.93	57.90^a^±7.48	62.00^a^±8.01	66.50^a^±8.59
***P. damnosus***	4.50^a^±0.58	5.00^a^±0.65	5.88^a^±0.76	6.55^a^±0.85	6.90^a^±0.89	7.00^a^±0.90	7.50^a^±0.97
***P. pentosaceus***	19.05^a^±2.46	21.00^a^±2.71	23.65^a^±3.05	26.65^a^±3.44	30.10^a^±3.89	30.00^a^±3.87	32.25^a^±4.16

Note: ^a-g^ Means with different superscripts within row are significantly different (P< 0.05).

According to Ouoba *et al.*[30], *L. acidophilus L. fermentum,* and *L. rhaminosus* contained positive amplicons of resistance genes encoding aminoglycoside (aph(3′)-III, aadA, aadE) and tetracycline tet (S), and hence they had higher prevalence of phenotypic resistance for aminoglycoside. This can be attributed to intrinsic resistance of different species since none of these bacteria were resistant to penicillin (first generation β- lactam). These organisms have the potential to act as reservoir of antimicrobial resistant genes and which have potential to be transferred to other bacteria. Moreover, intrinsic resistance to vancomycin was confirmed for *L. paracasei, L. salivarius* and *L. plantarum,* and *L. salivarius* was also typically resistant to erythromycin [31]. In other study, *L. salivarius* had the highest resistance to kanamycin and neomycin, but demonstrated the lowest resistance to penicillin [32]. *L. paracasei* subsp *paracasei* was usually used as probiotic in lactic acid fermented food for human consumption [33].

Vankerckhoven *et al*. [34] reported that *L. paracasei* 8700:2 strain was not resistant to antibiotic. However, *L. paracasei* subsp *paracasei* was found to be more resistant against nalidixic acid and sulphamethozole as compared to other antibiotics as found in this study. Furthermore, *L. plantarum* was sensitive to all the tested antibiotics except nalidixic acid, gentamycin, sulphamethoxazole, kanamycin and streptomycin. In contrast, Toomey *et al*. [35] claimed that *L. plantarum* harbored erm (B) and msrA/B genes, and tet (M) gene that resistant to erythromycin and tetracycline, respectively. Additionally, it is also intrinsically resistant to vancomycin. Similar trend was also found in *Lc. lactis* except for sulphamethoxazole in current study. Tetracycline and erythromycin-resistance genes were found in *Lc. lactis* and this microbe is normally representing the fermenting microflora of typical Italian traditional cheese Mozzarella di Bufala Campana [36]. It has been reported that *L. fermentum 1, Lc. lactic* subsp*. lactic 1, L. paracasei* subsp*. paracasei 1, L. rhamnosus* and *Lc. lactic* subsp*. lactic 2* isolated from raw poultry meat were resistant to polymycin B (PB 100), trimethoprim (TM 5), tetracycline (TE 30), oxacillin (OX 1), kanamycin (K 30), erythromycin (E 15), gentamycin (CN 10), ciprofloxacin (CIP 5) and cephalothin (CL 30) [37].

## Conclusions

In conclusions, many species of LAB were found in the faecal samples of broiler chicken. Each species of LAB has different inhibitory zone related to antibiotic susceptibility. These results indicate that LAB may develop resistance against antibiotic that may result from the horizontal transferring of resistant gene to other microflora in the gut. The development of resistance to antibiotic can be attributed to long term usage of antibiotic as therapeutics and growth promoter. Thus, it is important and essential to advise the farmer a proper way of antibiotic use as therapeutic and growth promoter agents. Additionally, supplementation of unknown source of probiotic as feed additive needs to be monitored closely in animal feeding.

## Competing interests

The authors declare that they have no competing interests.

## Authors’ contributions

NS and DWC conceived the idea and carried out the antibiotic resistance studies and initial analyses on collected data. NS has drafted the manuscript. HLF involved in experimental design, data acquisition, analyses and interpretation and revising the manuscript. TCL involved in conceptual the experimental design, data acquisition, analyses and interpretation of data, and revising the manuscript. RAR involved in acquisition of data and revising the manuscript. All authors read and approved the manuscript.

## References

[B1] Madden D Antibiotic resistance in *Escherichia coli A* practical investigation of bacteria conjugation National Centre for Biotechnology Education (NCBE): the University of Reading 2009

[B2] WHO ISDA Antimicrobial resistance Bad bug, no drugs as antibiotic discovery stagnates a public health crisis advance a review Official journal of the Australian Society for Microbiology Inc 2007 28 152

[B3] Scott KP The role of conjugative transposons in spreading antibiotic resistance between bacteria that inhabit the gastrointestinal tract Cell Mol Life Sci 2002 59 2071 2082 10.1007/s000180200007 12568333 PMC11146109

[B4] Schjørring S Krogfelt KA Assessment of bacterial antibiotic resistance transfers in the gut Int J Microbiol 2011 doi:10.1155/2011/312956 10.1155/2011/312956 PMC3034945 21318188

[B5] Loh TC Lee TM Foo HL Law FL Rajion MA Effects on growth performance, faecalmicroflora and plasma cholesterol after supplementation of spray-dried metabolite to postweaning rats Czech J Anim Sci 2008 54 10 16

[B6] Loh TC Chong SW Foo HL Law FL Growth performance and fecal microflora of rats offered metabolites from lactic acid bacteria J Appl Anim Res 2009 34 61 64

[B7] Thanh NT Loh TC Foo HL Hair-Bejo M Azhar BK Effects of feeding metabolite combinations produced by *Lactobacillus plantarum*on growth performance, faecal microbial population, small intestine villus height and faecal volatile fatty acids in broilers Br Poult Sci 2009 50 298 306 10.1080/00071660902873947 19637029

[B8] Loh TC Thanh NT Foo HL Hair-Bejo M Azhar BK Feeding of different levels of metabolite combinations produced by*Lactobacillus plantarum*on growth performance, faecalmicroflora, volatile fatty acids and villi height in broilers Anim Sci J 2010 81 205 214 10.1111/j.1740-0929.2009.00701.x 20438502

[B9] Choe DW Loh TC Foo HL Hair-Bejo M Awis QS Egg production, faecal pH and microbial population, small intestine morphology, and plasma and yolk cholesterol in laying hens given liquid metabolites produced by Lactobacillus plantarum strains Br Poult Sci 2012 53 106 115 10.1080/00071668.2012.659653 22404811

[B10] Thu TV Loh TC Foo HL Yaakub H Hair-Bejo M Effects of liquid metabolite combinations produced by*Lactobacillus plantarum*on growth performance, faeces characteristics, intestinal morphology and diarrhoea incidence in postweaning piglets Trop Anim Health Prod 2011 43 69 75 10.1007/s11250-010-9655-6 20632092 PMC2995859

[B11] Gueimonde M Sánchez B de Los Reyes-Gavilán CG Margolles A Antibiotic resistance in probiotic bacteria Front Microbiol 2013 4 202 doi:10.3389/fmicb.2013.00202 23882264 10.3389/fmicb.2013.00202 PMC3714544

[B12] Egervärn M Roos S Lindmark H Identification and characterization of antibiotic resistance genes in *Lactobacillus reuteri* and *Lactobacillus plantarum* J Appl Microbiol 2009 17 1658 1668 19457037 10.1111/j.1365-2672.2009.04352.x

[B13] Hamilton-miller JMT Shah S Vancomycin susceptibility as an aid to the identification of lactobacilli Let ApplMicrobiol 1998 26 153 154 10.1046/j.1472-765x.1998.00297.x 9569701

[B14] Danielsen M Wind A Susceptibility of Lactobacillus spp. to antimicrobial agents Int J Food Microbiol 2003 82 1 11 10.1016/S0168-1605(02)00254-4 12505455

[B15] Ammor MS Flórez AB Van Hoek AHAM de Los Reyes-Gavilán CG Aarts HJM Margolles A Mayo B Molecular characterization of intrinsic and acquired antibiotic resistance in lactic acid bacteria and *bifidobacteria* J Mol Microbiol Biotechnol 2008 14 6 15 10.1159/000106077 17957105

[B16] Korhonen JM Danielsen M Mayo B Egervarn H Axelsson L Huys G Antimicrobial susceptibility and proposed microbiological cut-off values of lactobacilli by phenotypic determination Int J Probiot Prebiot 2008 3 257 268

[B17] Mayrhofer S van Hoeck AHAM Mair C Huys G Arts HJM Kneifel W Antibiotic susceptibility of members of the Lactobacillus acidophilus group using broth microdilution and molecular identification of their resistance determinants Int J Food Microbiol 2010 144 81 87 10.1016/j.ijfoodmicro.2010.08.024 20888656

[B18] Foo HL Loh TC Law FL Lim YZ Kufli CN Gulam R Effects of feeding *Lactobacillus plantarum* I-UL4 isolated from Malaysian Tempeh on growth performance, faecal flora and lactic acid bacteria and plasma cholesterol concentrations in postweaning rats Food Sci Biotechnol 2003 4 403 408

[B19] Foo HL Loh TC Lim YS Syukriyah MH Kufli CN Law FL Effect of fermented fruits on growth performance, shedding of Enterobacteriacea and Lactic Acid Bacteria and plasma cholesterol on rats Pakistan J Nut 2003 2 228 233

[B20] Bauer AW Kirby WMM Sherris JC Turck M Antibiotic susceptibility testing by a standardized single disk method Am J Clin Pathol 1966 45 493 496 5325707

[B21] SAS Institute SAS/STAT User’s Guide, Version 6 1999 5 Cary, NC: SAS Institute Inc

[B22] Hilmi HTA Surakka A Apajalahti J Saris PEJ Identification of the most abundant Lactobacillus species in the crop of 1- and 5-week-old broiler chickens Appl Environ Microbiol 2007 73 67 73 10.1128/AEM.01128-07 PMC2168157 17933935

[B23] Kaktcham PM Zambou NF Tchouanguep FM El-Soda M Choudhary MI Antimicrobial and safety properties of lactobacilli isolated from two Cameroonian traditional fermented foods Sci Pharm 2012 80 189 203 10.3797/scipharm.1107-12 22396914 PMC3293358

[B24] Lavanya B Sowmiya S Balaji S Muthuvelan B Screening and characterization of lactic acid bacteria from fermented milk Br J D Sci 2011 2 5 10

[B25] Tyler LH Roberta JW Christian M Potent small molecule suppression of oxacillin resistance in methicillin resistant *Staphylococcus aureus* Angew Chem Int Ed 2012 51 11254 11257 10.1002/anie.201206911 PMC3829614 23047322

[B26] Gilchrist MJ Greko C Wallinga DB Beran GW Riley DG Thorne PS The potential role of concentrated animal feeding operations in infectious disease epidemics and antibiotic resistance Environ Health Perspect 2007 115 313 316 17384785 10.1289/ehp.8837 PMC1817683

[B27] Bassam YK *Leuconostoc mesenteroides* cause Nosocomial UTI at a tertiary care center in North India J Thi-Qar Univer 2011 4 23 34

[B28] Sieo CC Norhani A Tan WS Ho YW Plasmid profiling and curing of *Lactobacillus* strains isolated from the gastrointestinal tract of chicken J Microbiol 2005 43 251 256 15995642

[B29] Fukao M Tomita H Yakabe T Nomura T Ike Y Yajima N Assessment of antibiotic resistance in probiotic strain *Lactobacillus brevis* KB290 J Food Protect 2009 72 1923 1929 10.4315/0362-028x-72.9.1923 19777895

[B30] Ouoba LII Lei V Jensen LB Resistance of potential probiotic lactic acid bacteria and bifidobacteria of African and European origin to antimicrobials: Determination and transferability of the resistance genes to other bacteria Int J Food Microbiol 2008 121 217 224 10.1016/j.ijfoodmicro.2007.11.018 18063151

[B31] Blandino G Milazzo I Fazio D Antibiotic susceptibility of bacterial isolates from probiotic products available in Italy Microbial Ecolo Health Dis 2008 20 199 203 10.1080/08910600802408111 17201095

[B32] Majhenič AC Matijašič BB Antibiotics influence on lactic acid bacteria inhabiting gastrointestinal tract Mljekarstvo 2001 51 119 134

[B33] Sigrid M Angela HAM Christiane M Geert H Henk JM Wolfgang K Konrad JD Antibiotic susceptibility of members of the *Lactobacillus acidophilus* group using broth micro dilution and molecular identification of their resistance determinants Int J Food Microbiol 2010 144 81 87 10.1016/j.ijfoodmicro.2010.08.024 20888656

[B34] Vankerckhoven V Geert HH Vancanneytc M Klared CVI Romonde MB Entenzaf JM Moreillonf P Windg RD Knolg J Wiertzh E Poti B Vaughanj EE Kahlmeterl KG Gossensa H Biosafety assessment of probiotics used for human consumption: recommendations from the EU-PROSAFE project Tren Food Sci Technol 2008 19 102 114 10.1016/j.tifs.2007.07.013

[B35] Toomey N Bolton D Fanning S Characterization and transferability of antibiotic resistance genes from lactic acid bacteria isolated from Irish pork and beef abattoirs Res Microbiol 2010 161 127 135 10.1016/j.resmic.2009.12.010 20074643

[B36] Devirgiliis C Barile S Caravelli A Coppola D Perozzi G Identification of tetracycline and erythromycin resistant Gram- positive cocci within the fermenting microflora of an Italian dairy food product J Appl Microbiol 2010 109 313 323 20092542 10.1111/j.1365-2672.2010.04661.x

[B37] Lengkey HAW Balia RL Togoe I Taşbac BA Ludong M Isolation and identification of lactic acid bacteria from raw poultry meat Biotechnol Anim Hus 2009 25 1071 1077

